# Beyond Thriving Cities and Declining Rural Areas: Mapping Geographic Divides in Germany’s Employment Structure, 1993–2019

**DOI:** 10.1007/s11577-025-00992-4

**Published:** 2025-03-27

**Authors:** Gina-Julia Westenberger

**Affiliations:** Life Course and Inequality Research Centre (LIVES), Institute of Social Sciences, Université de Lausanne, 1015 Lausanne, Switzerland

**Keywords:** Spatial inequality, Labor market, Urban–rural divide, Left-behind regions, Occupational change, Räumliche Ungleichheit, Arbeitsmarkt, Stadt-Land-Unterschiede, Abgehängte Regionen, Beschäftigungswandel

## Abstract

This article assesses the popular thesis of growing regional inequality and urban–rural divides for Germany, focusing on the quality of employment opportunities. Drawing on a 2% sample of individuals registered in the German social security system over the period 1993–2019, we examine the level and evolution of employment opportunities along three established geographic divides—urban–rural, east–west, and north–south—at the small-scale level of districts (330 *Kreisregionen*). Occupation groups were ranked by median wage and assigned to quintiles to trace whether different districts experience diverging changes in their occupation structures. Our findings confirm significant urban–rural divides in the quality of employment opportunities, as upgrading has been much stronger in urban districts. Yet the differences we observe on an aggregated German level are also influenced by north–south and east–west divides, as these divides partially align. While some smaller cities and urban districts, mostly in southern Germany, have seen an above-average increase in high-wage jobs, most eastern German districts and many northwestern districts are still struggling to catch up with national upgrading trends. Our study thus shows that geographic polarization in Germany goes beyond simple urban–rural divides. Moreover, it demonstrates how examining the quality of employment opportunities provides long-term and spatially detailed indicators for studying geographic divides that capture a tangible aspect of regionally diverging life chances.

## Abstract (de)

In der vorliegenden Untersuchung wird die populäre These einer zunehmenden regionalen Ungleichheit und eines Stadt-Land-Gefälles für Deutschland einer kritischen Betrachtung unterzogen. Der Fokus unserer Analyse liegt dabei auf der Qualität der Beschäftigungsmöglichkeiten. Die Untersuchung basiert auf einer 2 %-Stichprobe von Personen (SIAB), die von 1993 bis 2019 im deutschen Sozialversicherungssystem registriert waren. Anhand dieser Daten werden das Niveau und die Entwicklung der Beschäftigungschancen entlang dreier etablierter geografischer Trennlinien – Stadt-Land, Ost-West und Nord-Süd – auf der kleinräumigen Ebene von 330 Kreisregionen analysiert. Die Berufsgruppen werden nach ihrem Medianlohn Quintilen zugeordnet, um festzustellen, ob verschiedene Kreise unterschiedliche Veränderungen in ihrer Berufsstruktur erfahren haben. Unsere Ergebnisse bestätigen ein deutliches Stadt-Land-Gefälle bei der Qualität der Beschäftigungsmöglichkeiten, da städtische Kreise einen deutlich stärkeren Zuwachs an gutbezahlten Arbeitsplätzen verzeichnen konnten. Die auf der aggregierten deutschen Ebene beobachteten Unterschiede werden jedoch auch durch das Nord-Süd- und Ost-West-Gefälle beeinflusst, da diese geografischen Polarisierungslinien sich teilweise überschneiden. Während einige kleinere Städte und städtische Kreise, vor allem in Süddeutschland, einen überdurchschnittlichen Anstieg an gutbezahlten Arbeitsplätzen verzeichnen konnten, haben die meisten ländlichen und städtischen Kreise im Osten und viele im Nordwesten weiterhin Schwierigkeiten, mit nationalen Trends mitzuhalten. Damit zeigt diese Studie, dass die geografische Polarisierung in Deutschland über ein einfaches Stadt-Land-Gefälle hinausgeht. Zudem stellt sie die Qualität von Beschäftigungsmöglichkeiten als langfristigen und räumlich detaillierten Indikator für die Analyse geografischer Unterschiede vor. Dieser Ansatz ermöglicht die Erfassung eines greifbaren Aspekts regional divergierender Lebenschancen.

## Introduction

The rise of populist parties and movements in Western nations over the last decade has renewed interest in geographic divides. It has been argued that changes in the economic structure caused by globalization, deindustrialization, and the shift to the knowledge economy have led to the economic decline of some regions, especially rural ones, while others, especially urban regions and large cities, are experiencing greater economic prosperity (Rodríguez-Pose 2018). In Germany, the recent success of the right-wing party Alternative for Germany (AfD) was interpreted as reflecting the division of regions into urban winners of globalization and left-behind rural regions. However, empirical evidence is mixed as to whether there is a growing divide between urban and rural districts in Germany. While some studies suggest that there is a more favorable development of urban districts (Belitz et al. 2019; Südekum et al. 2017), others contest that rural districts have been left behind (Oberst et al. 2019; Röhl 2018). Several studies find regional differences in economic output to be constant or even declining (Braml and Felbermayr 2018; Küpper and Peters 2019). To some extent, this divergence may be due to broad indicators such as growth in gross domestic product (GDP) per capita and specific, often short, time periods.

Our paper contributes to this debate by investigating whether urban and rural districts in Germany have diverged in terms of the quality of employment opportunities. We focus on the quality of employment opportunities because, for most people, they are a critical determinant of the life chances a place can offer them. Thus, we examine whether we can observe an urban–rural divide among German districts regarding the long-term change in the quality of their employment opportunities. Additionally, we analyze whether urban–rural divides in the employment structure are consistent across two other well-established lines of geographic polarization in Germany, namely east–west and north–south divides, and weigh these against each other. To fully grasp regional heterogeneity, we also map long-term changes in high-paying jobs for each of the 330 districts in our study.

Our analysis exploits a particularly rich dataset containing the labor market biographies of a 2% representative sample of all individuals registered in the German social security system (SIAB). This provides up to half a million observations per year for 330 districts (*Kreisregionen*) and allows us to run a more detailed spatial analysis than most studies on this topic (e.g., Rosés and Wolf 2021). To examine the differences in employment opportunities between urban and rural districts, we use the job skill method developed by Wright and Dwyer (2003). This method involves ranking occupations by median income and dividing them into quintiles of equal size at the start of the observation period. This allows us to observe how the composition of employment opportunities in a district changed between 1993 and 2019.

Our findings show significant divergences between urban and rural districts in Germany when it comes to the quality of their employment opportunities. Although employment change follows a similar trend of occupational upgrading across all types of districts, it is far stronger in more urban districts. This lends support to the claim of thriving urban areas and left-behind rural areas. Yet we observe that the clear-cut differences in employment change between urban and rural regions do not fully persist within different larger regions of Germany. While some smaller cities and urban districts, mostly in southern Germany, have seen a growth in good employment possibilities that was much higher than the national average, there are urban and rural districts, mostly in eastern and northern Germany, that do not follow national upgrading trends. By showing both the change in and actual structure of inequalities among districts, this article improves our understanding of geographic differences in employment opportunities and thus contributes to the broader debate on spatial inequality and its political consequences (Dijkstra et al. 2020; Gordon 2018).

## The Structure of Regional Economic Disparities in Germany

Regional divides based on the unequal distribution of economic opportunities within Western Europe have reemerged over the past three decades after a period of regional convergence from the 1950s to the 1970s (Rosés and Wolf 2021). During this period, the manufacturing economy enabled economic activity to spread more evenly across regions. Mature technologies such as car manufacturing required a lower concentration of skills and knowledge, which allowed a diffusion throughout the economy and thus led to deglomeration (Kemeny and Storper 2020). The fundamental changes in the economic structure caused by globalization, deindustrialization, and the subsequent transition to the service economy from the 1980s onward reversed this trend. The new technologies required a closer exchange of knowledge and resources, thus rewarding the clustering of firms and skilled workers in the same location—in most cases, densely populated urban areas (Kemeny and Storper 2020).

Places with a higher population density benefit from short distances that facilitate the exchange of goods and the supply of specialized business services, as well as opportunities for knowledge spillovers (Krugman 1991; Moretti 2013). Besides these agglomeration effects, large cities benefit from cultural factors, attracting the highly educated workforce required in knowledge-intensive industries and services (Diamond 2016; Florida 2017). Thick labor markets and skill abundance in large cities also lead to better assortative matching, making it easier for high-quality companies to find highly skilled workers, and vice versa (Dauth et al. 2022; Davis and Dingel 2020). As a result, urban areas depict greater productivity as well as higher employment growth, and they reward workers with higher wages than rural areas do, even when accounting for the higher cost of living (Kemeny and Storper 2020; Krugman 1991). However, employment change in large cities is not equally distributed: While the comparative advantage of knowledge-intensive industries in large cities resulted in an increase of high-skilled jobs in the service sector, the shift to the service economy also led to a decline of middle-skilled occupations, reallocating lower-skilled workers from production and office jobs to low-skilled services (Autor 2019).

In contrast, small cities, towns, and rural areas are seen as having experienced a deterioration of their economic conditions. Although these regions have never been on par with the dominant urban areas, they have been hit particularly hard by the loss of stable and well-paying jobs in the manufacturing sector (Davis et al. 2022). Although service-sector jobs were also created in rural areas, these jobs are more often low-paying or part-time with fewer benefits, as Brown and Schafft (2011) show for the United States. The lack of (good) employment opportunities has also led to high levels of out-migration, especially among the young and well-educated, trapping rural regions in a negative downward spiral (Johnson and Lichter 2019).

### The Urban–Rural Divide in Germany

This debate on regional polarization and the urban–rural divide is mainly dominated by research on the United States and, to a lesser extent, the United Kingdom. However, with the AfD’s growing success, a lively public debate is taking place in Germany about the extent to which the rise of the far right party has been driven by a growing urban–rural divide in economic opportunities (Bollmann and Kloepfer 2018). These debates have in turn inspired a lively discussion in the social sciences about whether we can observe an increasing dichotomy between prosperous urban regions and a declining countryside (Haffert 2022; Konietzka and Martynovych 2022, 2023; Reckwitz 2019). With regard to the structural underpinnings of this social and political debate, there is disagreement about the role and extent of the urban–rural divide in Germany. Of course, urban–rural divergences can be discussed from a variety of different angles, ranging from the quality of the infrastructure or public services to demographic indicators to economic measures such as productivity, median wages, and employment rates (Diermeier 2020; Fink et al. 2019; Küpper and Peters 2019). In this article, we consider the urban–rural divide from a more economic perspective, focusing in particular on the quality of the employment structure and labor markets.

A number of studies show significant economic disparities between urban and rural districts in Germany. German cities benefited from the growth of employment in the service economy, while some rural regions dependent on manufacturing suffered due to the availability of cheaper imported goods (Südekum et al. 2017). Furthermore, the average productivity in urban German regions is higher than in rural regions (Belitz et al. 2019; Küpper and Peters 2019). Better matching between high-quality workers and high-quality plants also leads to higher wages in Germany’s large cities (Dauth et al. 2022). Plus, a large share of the country’s top 10% of earners live in and around major cities, such as Hamburg, Munich, and Cologne (Immel and Peichl 2020).

Other studies contest this argument of thriving cities and poorly performing rural regions in Germany. Oberst et al. (2019), for instance, found that urban districts performed worse than semiurban and rural districts on their indicator for economic development, consisting of the regional unemployment rate, GPD per capita, purchasing power, and household debt. Also, the increasing concentration of population in cities is not reflected by an increasing concentration of GDP per capita in them (Röhl 2018). There are no clear urban–rural patterns in terms of employment growth either, particularly because some western German cities in the Ruhr region, such as Gelsenkirchen and Oberhausen, experienced a marked decline in employment (Südekum et al. 2017). In terms of changes in the occupation structure, small towns and villages have moved alongside the cities toward a postindustrial occupation structure, with growing shares of the new middle class and declining shares of the old nonmanual middle class (Konietzka and Martynovych 2022).

### Other Geographic Divides in Germany

One reason why the extent of the urban–rural divide in Germany is disputed is that with its decentralized and polycentric political and economic structure, the country presents a least-likely case for pronounced urban–rural divides. Economic activity is not confined to Germany’s metropolises, but Germany’s so-called hidden champions—small and little-known world market leaders—are located outside the metropolises in medium-sized and small towns (Lang and Vonnahme 2020). In addition, Germany has a well-established debate about geographic divides that are a product of its country-specific history. This mainly concerns the persisting differences between eastern and western German regions (Mau et al. 2024), as well as the north–south divide between the economically strong south and the economically weaker north (Lammers 2003).

The economic disparities between eastern and western German regions have deep-rooted historical origins, with some scholars arguing that productivity differences even predate the Second World War. At the time of reunification in 1990, eastern German regions exhibited markedly lower productivity, a consequence of decades of failed planned economy (Ritschl 1995). The privatization process of the formerly state-owned enterprises of the German Democratic Republic (GDR), managed by the Treuhand agency, proved to be particularly challenging, with most companies being sold at minimal prices or remaining unsellable, often failing to generate economic success (Roesler 1994). This drastic economic transformation precipitated mass unemployment in eastern regions, triggering substantial population loss, especially among young and skilled workers (Dienel 2004). Recent studies indicate progress in economic convergence, including reduced disparities in the unemployment rate, pension equalization, and demographic stabilization due to the attractiveness of bigger eastern cities such as Leipzig, Dresden, and Potsdam (Mau 2024). However, significant economic divides persist, for instance in wealth inequalities between citizens in the east and the west (Mau 2024).

A third significant line of geographic polarization in Germany is the north–south divide. Historically, the northern regions were more prosperous, as they dominated industrial production during the nineteenth century’s industrialization period, while the south remained predominantly agricultural. Since the 2000s, this pattern has, however, reversed, with southern regions, particularly the federal state of Bavaria, emerging as centers of innovation and economic growth (Lammers 2003; Wolf 2016). This transformation emerged from multiple interconnected factors, including post–World War II migration pressures to the south that necessitated industrial development. Mainly, the trend toward tertiarization from the 1970s and the rise of light industry significantly enhanced southern regions’ economic competitiveness. While the economy in the north of Germany was characterized by basic and heavy industry, the pronounced small and medium-sized business culture in the south facilitated rapid technological innovation, particularly in knowledge-intensive sectors such as microelectronics and automotive engineering. Additional favorable factors included strategic location policies, substantial investments in higher education, and a strong tradition of self-reliance rooted in the south’s agricultural organizational structure (Wolf 2016).

### Increasing or Decreasing Spatial Divides?

In addition to the structure of these geographic divides, another question is whether and how such geographic divides have changed in recent decades. Contrary to the increase in spatial inequality observed for other Western countries, several studies show that economic disparities between German districts have been declining or remained constant in the past two decades (Braml and Felbermayr 2018; Küpper and Peters 2019; Röhl 2018). For instance, regional GDP per capita became more evenly spread between 2000 and 2014 (Braml and Felbermayr 2018). Inequality among German districts has also decreased with regard to (household) incomes in the last two decades (Frieden et al. 2023; Fuest and Immel 2019; Immel and Peichl 2020). These trends are mainly driven by the catching-up of former East German districts following the reunification (Braml and Felbermayr 2018; Fuest and Immel 2019; Immel and Peichl 2020). Eastern districts have seen some of the fastest growth in GDP per capita, as growth and initial levels in GDP per capita are, on average, negatively correlated (Braml and Felbermayr 2018). However, when western German districts only are analyzed, growing regional polarization in GPD per capita is reported (Braml and Felbermayr 2018).

## Occupational Change and Regional Divides

Our paper introduces the quality of occupational change as an alternative way to study urban–rural disparities in Germany. Geographic divides are, of course, possibly characterized by a multitude of features, as the lively debate on the definition of left-behind places shows (Pike et al. 2023). Yet we believe that studying the quality of jobs offered by a district, and tracing how these employment opportunities have developed over time, adds an important piece to the puzzle. We are confident that this will also address some of the following concerns we raise about the current literature.

First, the indicators used by previous studies also impact their findings, especially the per capita indicators such as GDP per capita (Braml and Felbermayr 2018; Küpper and Peters 2019; Oberst et al. 2019). These are—as some authors have pointed out—sensitive to demographic developments and changes in the population size. For instance, out-migration of the population can lead to an improvement in economic indicators, while the influx of nonworking population groups, such as students into university cities, leads to a weakening. Neither, however, reflects the actual economic opportunities of a district. This concern applies, for instance, to rural districts in eastern Germany that have seen a significant decline in their population size, which led in some cases to an increase in GDP per capita (Oberst et al. 2019). Whether this really reflects an improvement for the remaining population in this district is questionable.

Second, we believe that the indicators used by previous studies lack nuance about the economic situation of a region. For example, average wages do not include regionally differentiated costs of living, especially rental costs. This puts into perspective the higher incomes in large cities, where rental prices are also higher. Additionally, using general employment growth as an indicator (Südekum et al. 2017) does not provide any information on the quality of the employment opportunities created. For example, the promotion of a place as a tourist destination in one district and the establishment of a new high-tech company in another district could result in the same number of new jobs. Yet these jobs would be very different in terms of the wages paid or other benefits provided and would therefore have a markedly different impact on the lives of the people in that district.

While different indicators provide different aspects to research, we are confident that studying occupational change can add interesting insights to the debate on urban–rural divides and spatial inequalities and address some of the shortcomings of other indicators that we have discussed above. We propose studying (the development in) the quality of employment in a region because for a large majority of working-age individuals, the income generated by employment is the main source of earnings. Individual life chances are thus dependent on the availability of well-paying jobs and good labor markets: The more well-paying jobs are available in a district, the better the individual life chances in these districts should be. The expansion of high-quality employment also generates substantial multiplier effects within regional economies. Beyond the direct beneficiaries of good jobs, the increased disposable income of higher earners stimulates local service sectors and their support industries (Moretti 2013). Moreover, regional employment change and occupational upgrading may serve as tangible indicators of how the region one lives in is doing economically. A rich literature in political science has shown that these perceptions of local economic contexts affect political preferences and voting behavior (Backes and Müller 2024; Colantone and Stanig 2018; Dijkstra et al. 2020; Essletzbichler et al. 2018; Jennings and Stoker 2016; Patana 2020, 2022).

As discussed above, it is notoriously difficult to study the quality of labor markets while maintaining a certain simplicity of indicators. Therefore, we follow the long tradition of capturing the quality of labor markets by analyzing the evolution of employment in skill-based quintiles (Autor et al. 2008; Eurofund 2024; Goos and Manning 2007; Oesch 2013; Wright and Dwyer 2003). We believe that this approach allows us to fruitfully study employment change in regional labor markets and spatial disparities between different regional labor markets.

What expectations can be derived regarding the structure and evolution of urban–rural divides in Germany’s occupational structure? First, we anticipate significant disparities in the employment structure between urban and rural regions. Second, we also expect to find these clear urban–rural divides in the way the employment structure has changed over our study period. In particular, we expect that occupational upgrading—characterized by the growth in high-skilled employment—has been more pronounced in urban districts. While Germany’s overall employment transformation has followed a trajectory of widespread occupational upgrading (Oesch and Piccitto 2019), the knowledge-intensive jobs emerging in the expanding service sector (Dauth et al. 2017) are likely to be concentrated in larger cities and metropolitan areas. Contrary to more centralized countries such as France or the United Kingdom, where the economic activity is heavily concentrated in its capital cities, we expect that highly skilled jobs in Germany will be spread more evenly but will still be concentrated in more urban districts and not in the countryside. We account for this expectation of good employment opportunities in medium-sized cities by applying a fine-grained indicator for urbanity instead of a simple dichotomous distinction, as will be explained in the methods section of this paper. Furthermore, given that spatial inequality in Germany is also characterized by east–west and north–south divides, we also expect these divides to be reflected in the employment structure. The relative magnitude of these various geographic divides will be examined in our empirical analysis.

## Research Design

### Data and Sample

Our analysis is based on a sample of the Integrated Labor Market Biographies (SIAB),^1^ a unique dataset spanning three decades from 1993 to 2019 (Frodermann et al. 2021a; 2021b). The SIAB offers a 2% random sample of all individuals registered in the German social security system. The information transmitted to the social security system is, among other uses, used to calculate pensions and should therefore be very reliable data. The time period chosen was after the German reunification in order to allow comparisons between western and eastern districts. The starting point of the study results from the fact that 1993 is the first year in which social security data for the east are reliable according to the data owner. This leaves us with an observation window of three decades, providing a considerably longer period than previous research and also capturing the disruptions caused by the long postreunification recession in the 1990s (Oberst et al. 2019).

Because the SIAB is based on individuals enrolled in the public social security system, the analytical sample is limited to employees, excluding civil servants and the self-employed. This is an unfortunate limitation of the dataset. Nonetheless, our study covers the vast majority of the active labor force in Germany, with the share of employees ranging from 83.1% in 1993 to 81.9% in 2019 (Federal Statistical Office of Germany 2024). Although it would be of interest to examine the evolution of the occupations taken up by civil servants and the self-employed, we believe that the exclusion of these two groups would change our results most significantly if we were to see a marked increase in the number of people employed in these two groups. However, this is not the case, as the share of self-employed has remained relatively stable during the period of study (Online Appendix, Fig. A1).

In order to study the evolution of good employment opportunities, we further limited the analytical sample to people between 20 and 60 years of age who worked in jobs that were subject to social security contributions. Following Dauth and Eppelsheimer (2020), we transformed the daily spell structure of the SIAB into a yearly sample, with June 30 as the cut-off date for each year. Per individual, only one main job is kept, which is defined as the job with the highest daily wage. In our analytical sample, the number of observations ranges between 506,002 in 1993 and 540,613 in 2019.

Registration of employment is based on the location of the place of work, rather than the location of the residence of the respective employee. Regions are distinguished on the level of the 330 districts (*Kreisregionen*) provided by the SIAB. Although the number of these official administrative districts in Germany is 361, the SIAB data aggregate some smaller districts for data protection reasons, so that each aggregated unit contains at least 100,000 inhabitants. Still, this allows for a more detailed spatial analysis than most studies on this topic (see, for example, Rosés and Wolf 2021).

**Fig. 1:**
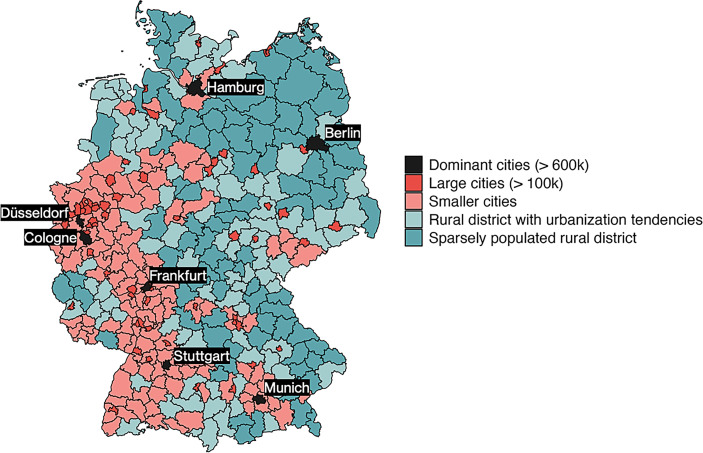
German districts according to their degree of urbanity/rurality (author’s depiction, using data by BBSR 2019)

The rurality or urbanity of a district is captured with a four-scale indicator^2^ (BBSR 2019): Based on the population share living in large and medium-sized cities and the general population density of a district, it distinguishes large urban areas with more than 100,000 inhabitants (*N* = 60), urban areas (*N* = 116), districts with urbanization tendencies (*N* = 77), and sparsely populated rural districts (*N* = 70). In addition, we added a fifth category for the largest metropolitan areas in Germany. These superstar cities are defined as the districts of the seven largest German metropolises (> 600,000 inhabitants): Berlin, Hamburg, Munich, Cologne, Frankfurt, Düsseldorf, and Stuttgart. This indicator, displayed in Fig. 1, distinguishes five types of places and thus allows for a more fine-grained analysis of types of districts than a simple dichotomous urban–rural indicator (e.g., Sixtus et al. 2019).

### Analytical Strategy

To trace changes in the employment structures of districts, we followed the job skill method of Wright and Dwyer (2003). We did so by ranking each of the 120 occupations classified by the German occupational classification KldB 1988 according to their earning potential in 1993. The earning potential was measured using the daily median wage^3^ of all men and women working full-time in this job, averaged over a 5-year period (1993–1997). For the calculation of the occupational median wages, we focused on full-time employment because the imprecise binary differentiation of part-time work in the data would underestimate wages in occupations with higher shares of part-time workers. We relied on wages to measure the skill requirements and quality of a job, as wages are seen as putting a price tag on bundles of skills required in a specific occupation. This allows for a more fine-grained distinction of skills than information on educational degrees alone. In this we follow other influential research on occupational change, such as that conducted by Autor (2019) and Oesch (2013). To validate our approach, we replicated our analyses by calculating mean years of education for each occupation with the information on educational attainment provided in the SIAB. Indeed, this correlates strongly with our wage-based skill measure (r = 0.76). All occupations were then rank-ordered according to their median earnings and divided into quintiles, each containing as close as possible to 20% of people working full- and part-time in 1993. This allocation of occupation groups to quintiles is illustrated in Table 1.

**Table 1:** Allocation of the 120 occupation groups to job-quality quintiles

Rank	Occupation group (KldB 1988)	Quintile
95–120	Managers, doctors, industrial foremen (…)	5
82–94	Social pedagogues, engine attendants, nurses (…)	4
47–81	Chemical plant workers, kindergarten teachers, police officers (…)	3
21–46	Masons, painters, office hands (…)	2
1–20	Shop assistants, hairdressers, cooks (…)	1

Another concern regarding the ranking of occupations by median wages might be the urban wage premium; i.e., occupations concentrated in larger cities have higher average earnings, overestimating their skill level and thus the wages offered to these occupations outside metropolitan areas. As a robustness check, we therefore calculated the occupational median wages by excluding all observations of people working in large urban areas (> 100,000 inhabitants). Ranking of the occupations by these median wages in 1993 indicates that the position of the 120 occupational groups in the quintiles correlates almost perfectly with the assignment obtained by including observations from cities (r = 0.947). This highlights the advantage of tracking the quintiles rather than focusing on actual earnings. While a physician’s and a hairdresser’s earnings may similarly differ depending on whether they work in Munich or two hours away in a smaller village in Bavaria, being a physician will be a better paid occupation than being a hairdresser in both locations.

To trace the changes in a district’s quality of the employment structure over time, we counted the share of people in a district working in a job belonging to the job-quality quintile we assigned it to for each year and then compared these shares between the years. We thus followed a descriptive approach to document regional changes in the occupational structure rather than to explain their causes. The main idea of the job skill method is to keep the allocation of occupations to quintiles constant over time, to observe how employment changes over time within each quintile. However, this requires an occupation scheme that is stable over time, which is not the case for the SIAB. Instead, the decision of the Federal Employment Agency to convert from the KldB 1988 to the KldB 2010 in 2011 led to a major break in the data (Fig. A2 in the Online Appendix). In order to avoid an inconsistency in the occupation ranking, we therefore calculated a second ranking, ranking the 125^4^ occupations classified by the KldB 2010 as described above and dividing them into quintiles, each of which contains as close as possible to 20% of people working full- and part-time in 2012. Both in 1993 and 2012, the bottom quintile contains occupations such as hairdressers, cooks, shop assistants, and cleaners, while the top quintile contains occupation such as physicians, engineers, IT specialists, and managers.

Due to this reranking, it is not sensible to directly compare quintile sizes in 1993 and 2019 if we are interested in a change rate, as the reranking results in slightly different initial quintile sizes.^5^ Therefore, we calculated the changes within an occupational scheme separately and then aggregated the rates of change as follows:

$$\Updelta _{2019-1993}=(E(Q)_{2010}-E(Q)_{1993})+(E(Q)_{2019}-E(Q)_{2012})$$

where E(Q)_2010_ and E(Q)_1993_ are the shares of employed persons in each quintile based on the 1993 ranking, and E(Q)_2019_ and E(Q)_2012_ are the shares of employed persons in each quintile based on the 2012 ranking. The primary focus of this paper will be on this change rate, although we will also resort to the current distribution of the job-quality quintiles for certain analyses. This refers to the proportion of individuals in each of the five job-quality quintiles in 2019, based on the 2012 occupation–income ranking. Because we use a reranking and thus two shorter-term change rates to calculate employment changes in the quintiles, our approach may lead to a rather conservative estimate of employment change compared to other studies that rely on a single ranking over the entire study period (Oesch and Piccitto 2019). Yet because the focus of our paper is mainly on how different (urban or rural) districts have developed over time in comparison to other (urban or rural) districts, this should be a minor issue.

## Results and Discussion

### Urban–Rural Divides

**Fig. 2:**
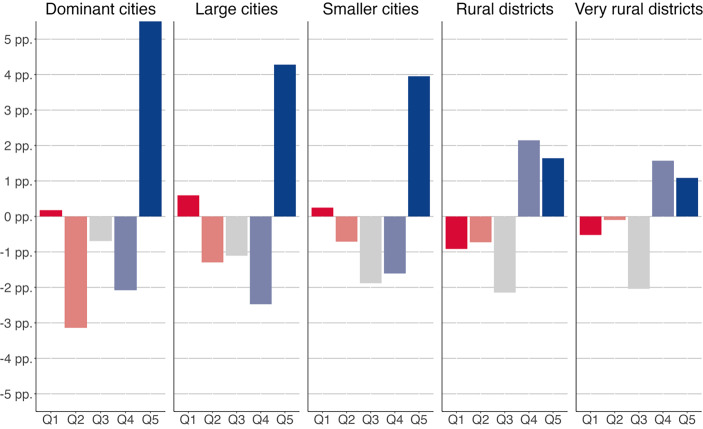
Relative employment growth across job-quality quintiles in different types of districts, Germany 1993–2019. This figure presents the relative change in the share of people working in each of the job-quality quintiles from 1993 to 2019. Because quintiles amounted to 100% at the beginning and end of the study, this figure shows *relative *and not *absolute *change in employment per type of district. *pp* percentage points

In line with our expectations, we find a clear difference between urban and rural districts in terms of changes in their employment structure. This can be seen in Fig. 2, which shows the relative employment growth in percentage points within the groups of urban and rural districts between 1993 and 2019. First, all types of German districts have experienced an upgrading of their employment structure, such that employment growth was highest in the well-paid jobs of the fifth (in more urban districts) and fourth (in rural districts) quintiles. This is in line with previous research highlighting occupational upgrading trends in Europe (Oesch and Piccitto 2019). However, the strength of upgrading is clearly related to a district’s degree of urbanity, as the upgrading of the employment structure is significantly stronger in urban than in rural districts. In the largest German cities, the increase in quintile five jobs was over four percentage points higher than in the most rural districts. So even in a decentralized economic system like Germany, with its “hidden champions” in more rural districts, the growth of particularly good jobs is greatest in the largest cities.

Another aspect clearly distinguishes urban districts from more rural ones: Rural districts have gained the most new jobs in the fourth quintile and slightly less in the fifth quintile, while employment growth in urban districts is more polarized. In addition to the strong increase in the top quintile, there has also been a small increase in very low-paying jobs in the bottom quintile in urban districts. This is in line with previous research on the slight polarization in urban areas in Germany (Dauth 2014) and is consistent with theories of polarized technological change (Autor and Dorn 2013).

**Fig. 3:**
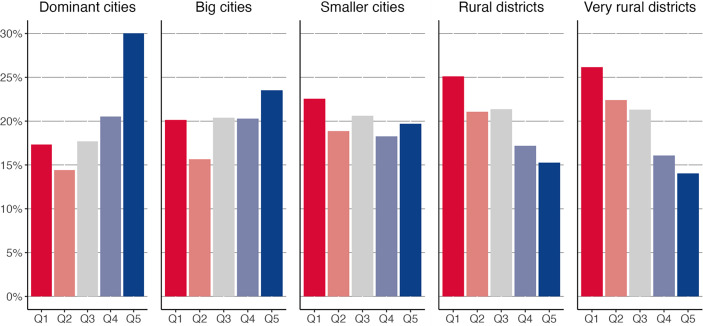
Employment across job-quality quintiles in different types of districts, Germany 2019. This figure shows the proportion of people employed in each job-quality quintile by type of district in 2019. The levels and distribution of the quintiles shown are based on our 2012 occupation–income ranking, using the KldB 2010 occupation code

So how is the quality of employment distributed between urban and rural districts if urban districts have experienced greater upgrading over the long term? Fig. 3 compares the employment in urban and rural districts in 2019^6^ across quintiles and highlights the marked differences between the different types of districts. In the largest cities, approximately one-third of jobs are in the top quintile, and another 20% are in the fourth quintile. Also in the big city districts, the highest quintile share is reached in the top quintile. Conversely, in the most rural districts, the proportion of jobs in the top quintile of total employment is less than 15%, which is half the proportion observed in the largest cities. Even if this share of good jobs has increased, the occupational structure in the smaller cities and in (very) rural districts is dominated by jobs in low-paid occupations, with the largest share of people working in very low-paid occupations in the first quintile. In contrast to previous studies (e.g., Südekum et al. 2017), we thus find clear differences in the employment structure of urban and rural districts, both in terms of their development over the past decades as well as in current employment opportunities.

The approach of this analysis is descriptive, and our data preclude causal inference about the causes of these different patterns of employment change. However, examining the correlation of quintile shares between 1993 and 2019 can provide insight into how initial labor market structures shape subsequent changes in regional employment opportunities. Surprisingly, the correlation of the employment shares in the respective quintiles is in most cases rather moderate, with a notable exception of stronger correlation for the region’s quintile five shares (Tables A1 and A in the Online Appendix). When considering correlations separately by types of urban and rural regions, we see that quintile sizes in the biggest cities correlate quite strongly between 1993 and 2019. However, this category is composed by the small and likely homogeneous number of Germany’s seven biggest cities. All three types of urban regions share a high correlation of quintile five and quintile one employment shares, whereas the association between quintiles two to four shares is much weaker or even negligible in bigger cities and urban regions. In the (very) rural regions, all correlations between initial employment shares and quintile employment in 2019 are much weaker, notably also for employment in the top quintile. Thus, although there is some evidence—particularly with regard to well-paid employment in urban areas—that the trajectories of employment opportunities in a region are influenced by previous conditions, these analyses mainly highlight that there appears to be a diversity in trajectories.

### Rural–Urban Divides in Northwest, Eastern, and Southern Germany

Urban–rural divides can be cross-cutting or align with other geographic divides in a country. In Germany, the historic political east–west and the long-run economic divide between north and south form the two established lines of geographic polarization that might also be captured by our description of urban–rural divides, as urban and rural districts are not evenly spread across the country (Fig. 1). For example, most of the very rural districts are located in the east of the country, while an urban strip stretches from the Ruhr area via Frankfurt to the south of Baden-Württemberg. This could potentially lead to overestimation of the extent of the urban–rural divide, as it subsumes further geographic cleavages. It is therefore essential to, at the very least, relate our analysis to east–west and north–south differences.

In this second part of the analyses, we therefore analyzed whether urban–rural divides in the quality of employment change were consistent *across different parts of Germany. *For this purpose, we first replicated our earlier analyses on the quality of employment change across urban and rural districts separately for three larger regions of Germany. Because the east–west and the north–south divides overlap geographically, we simplified the analysis by restricting it to three regions: the northwest, the south (districts in the federal states of Bavaria and Baden-Württemberg), and the east (all former GDR districts).

**Fig. 4:**
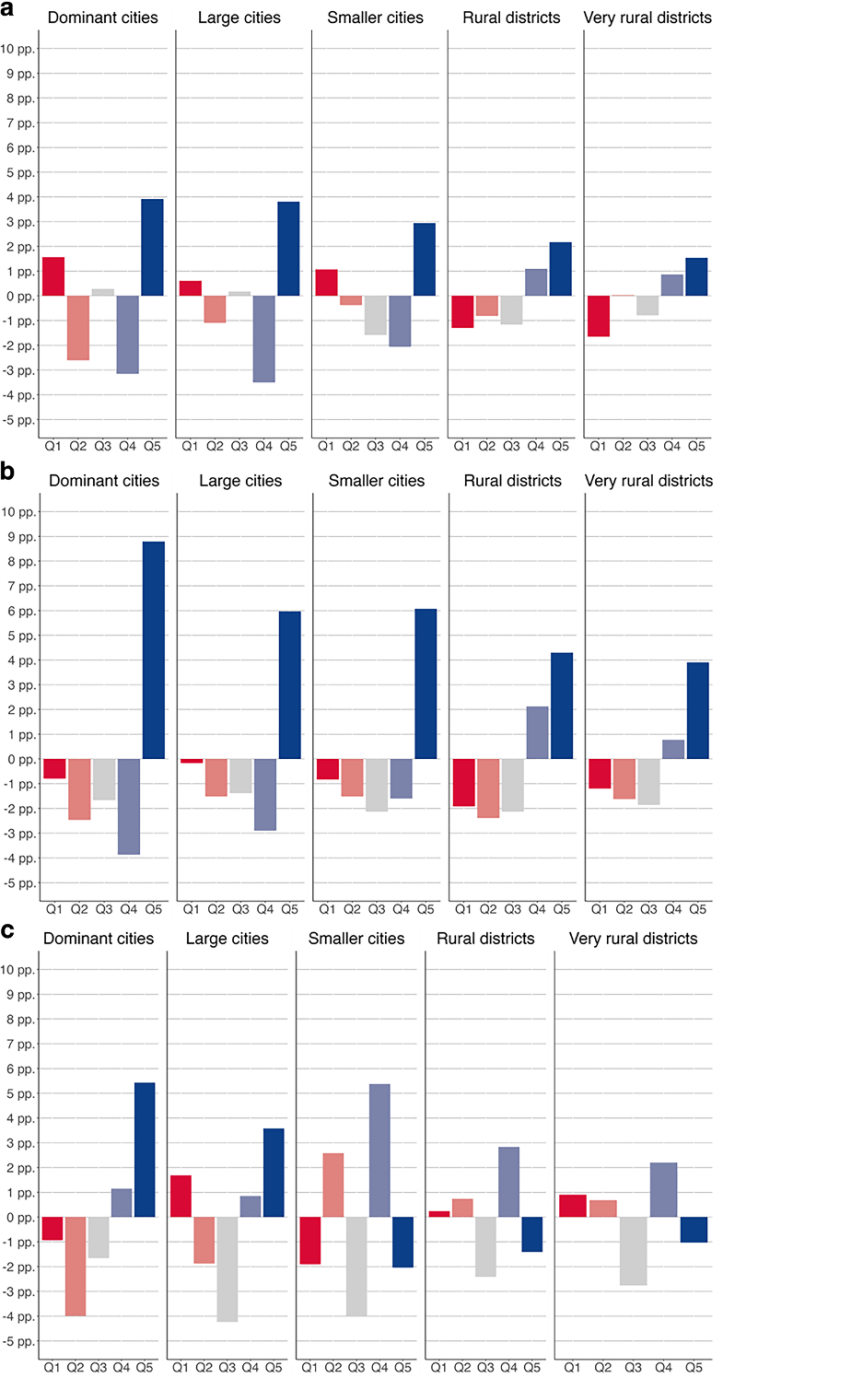
Relative employment growth across job-quality quintiles in different types of districts across northwestern, southern, and eastern Germany, 1993–2019: **a** northwest, **b** south, **c** east. These figures depict the percentage point (pp) change between 1993 and 2019 in the share of people employed in each job-quality quintile in the northwest, south, and east of Germany. The northwest includes districts in the federal states of Bremen, Hamburg, Hesse, Lower Saxony, North Rhine-Westphalia, Schleswig-Holstein, Rhineland-Palatinate, and Saarland. The south includes districts in the federal states of Bavaria and Baden-Württemberg, and the east includes districts in the federal states of Brandenburg, Mecklenburg-Western Pomerania, Saxony, Saxony-Anhalt, and Thuringia

Employment change in northwestern districts most closely resembles the patterns we observed across urban and rural districts in Germany in general (Fig. 4a). Across all types of districts, employment growth was strongest in quintile five, yet the strength of this upgrading is clearly related to the urbanity of a district. Again, the upgrading in urban districts was accompanied by distinct employment decline in the middle quintiles two to four and a growth of jobs in the bottom quintile. Notably, this slight polarization of the employment structure is more pronounced in the biggest cities of Cologne, Düsseldorf, Frankfurt, and Hamburg and the smaller city districts than in larger cities.

In southern districts, the upgrading of the employment structure has been strong across urban and rural regions, making the urban–rural divide less pronounced (Fig. 4b). In Germany’s two largest southern cities, Munich and Stuttgart, the strength of employment upgrading is significantly higher than in other major German cities. And it is as high in the larger cities and smaller city districts as it is in Germany’s dominant cities. Even in (very) rural regions, the increase in highly paid employment was only about 1.5 percentage points lower than in the more urban districts. With approximately four percentage points, the upgrading in the (very) rural regions of the south was much stronger than in rural districts in other parts of the country, and even higher than in the larger cities in the northwest and east.

In contrast to the other regions of Germany, the eastern districts have experienced a markedly different pattern of employment change (Fig. 4c). Unlike in the northwest and south, employment change did not follow a similar pattern—yet to varying degrees—across urban and rural regions. While employment change in the major city in the east, Berlin, has followed a strong upgrading pattern similar to those in Munich and Stuttgart, other large cities in the east have experienced employment polarization, with a notable increase of quintile five and one employment. In smaller cities and (very) rural districts, an increase in quintile four employment was accompanied by an increase in the bottom and second quintile. The (very) rural districts of eastern Germany are the only rural districts in this aggregated analysis where we observe polarized employment change instead of a clear upgrading pattern.

In sum, these analyses show that the clear-cut differences in employment change between urban and rural regions we find on an aggregated level do not fully persist across different larger regions of Germany. This supports the notion that the urban–rural divide at least partially aligns with other established lines of geographic polarization.

Our descriptive analyses are, however, constricted to further disentangle this alignment of geographic divides. Still, we would like to gain a preliminary understanding of the relative significance of each of these divides for regional employment change. We therefore employed a series of simple linear regression models that allowed us to compare the impact of different geographic divides on our variable of interest. For these models, we combined the three urban categories (dominant, large, and small cities) and the two rural categories to create a binary indicator that could then be compared with the three categories for the geographic location. Effects are displayed using urban, western, and northern districts as the respective reference categories. For the dependent variables, we focus on the change and level in top quintile employment, as we believe that these high-paying jobs are critical for defining life chances in a district.

The results of these models, presented in Table 2, indicate that the significance of the geographic divides varies depending on whether the focus is on the development of employment or on its distribution at present time. The change in the top quintile employment appears to be more significantly influenced by the broader regional divides than by urban–rural differences (model 1). Compared to urban regions, rural regions are associated with a significantly lower upgrading rate. However, this effect is smaller than the ~2.3 percentage point increase in top quintile employment change associated with the southern districts and the ~2.8 percentage point decrease associated with the eastern districts.

**Table 2:** Comparison of the effects of urban–rural, north–south, and east–west divides on top quintile employment in Germany

	Change 1993–2019 in top quintile employment (in percentage points)	Share 2019 in top quintile employment (in %)
	(1)	(2)
Rural districts (binary)	−1.23^***^	−5.88^***^
	(0.32)	(0.55)
Eastern districts	−2.82^***^	0.80
	(0.44)	(0.75)
Southern districts	2.33^***^	3.62^***^
	(0.34)	(0.58)
Constant	3.16^***^	19.00^***^
	(0.23)	(0.40)
Observations	330	330
R^2^	0.36	0.33
Adjusted R^2^	0.35	0.32
Residual standard error	2.68 (df = 326)	4.62 (df = 326)
F statistic	60.28^***^ (df = 3; 326)	53.68^***^ (df = 3; 326)

The table presents the regression coefficients for the two regression models testing the association of urban–rural, north–south, and east–west divides with the change (1993–2019) and 2019 share in top quintile employment. The reference categories for the binary independent variables are urban/western/northern districts. The units of analysis are the 330 German districts

^*^p < 0.1, ^**^p < 0.05, ^***^p < 0.01

The rurality of a district, however, has a greater impact than a district’s geographic location on the share of top quintile employment (model 2). While the share of Q5 employment is estimated to be 5.9% lower in rural than urban districts, southern districts are associated with a ~ 3.6% higher share of top quintile employment. It is noteworthy that districts in eastern Germany are estimated to not have a significantly lower share of quintile five employment when accounting for differences in the number of rural districts in the east and west of Germany. Consequently, the current life chances and good employment opportunities in a region appear to be more closely linked to its urbanity, while the northwest/east/south geographic divides in Germany had a greater impact on the trajectory of a region’s labor market opportunities. To test whether the effect of the urban–rural divide depends on the geographic location of a region, we also included an interaction term in both models (Table A3 in the Online Appendix). However, there is no significant interaction effect between rurality/urbanity and location, either for the change in or the proportion of well-paid employment.

### Trajectories of Single Districts

As the final step of our analysis, we aim to illustrate the findings from our previous aggregated analyses in greater depth by mapping employment levels and shares for each of the 330 individual districts. This allows us to explore regional heterogeneity as well as the aforementioned overlaps between the geographic divides in more detail. Because a presentation in map format is inherently limited in its ability to present several variables in a comprehensible manner, we will once again focus on top quintile employment. We also concentrate our description on the districts with the largest increases or decreases, as well as the highest and lowest levels of quintile five employment.

**Fig. 5:**
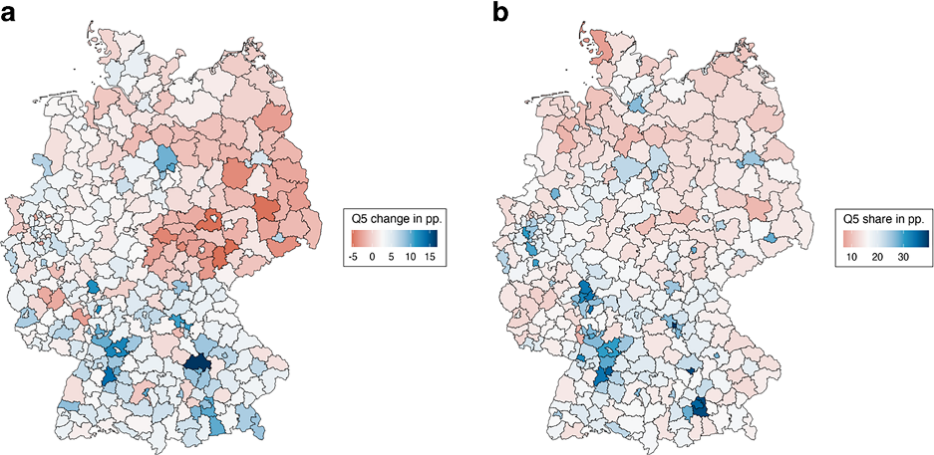
Employment change within districts and employment level in top quintile. **a** Change in top quintile employment, 1993–2019, representing the percentage point change between 1993 and 2019 in the share of people employed in the top quintile for each of the 330 German districts.** b** Share of top quintile employment, 2019, representing the share of people employed in the top quintile in 2019. In both figures, regional values are colored in relation to the national average, with red representing values below and blue representing values above the national average

Figure 5a maps how employment in quintile five evolved in each of the 330 districts between 1993 and 2019 relative to the national average change (which increased by three percentage points). In (dark) red districts, the share of employees in quintile five decreased compared to the national average. In (dark) blue districts, this share increased. The three districts with the strongest job growth in quintile five are located in the south: Eichstätt (+17 percentage points) in Bavaria and Böblingen (+14 percentage points) and Heilbronn (+13 percentage points) in Baden-Württemberg. The three districts with the highest decline in good jobs are all located in eastern Germany: Saalekreis in Lower Saxony (−5 percentage points), Greiz/Gera in Thuringia (−5 percentage points), and Elbe-Elster in Southern Brandenburg (−5 percentage points). Again, this highlights the presence of strong east–west and north–south divides. Districts that have seen an increase in highly paid jobs are mainly located in the south and, to a lesser extent, in the west of Germany. Conversely, districts with a declining share of highly paid jobs are mostly found in the east and north, as well as some districts in the west such as the Ruhr area and Rhineland-Palatine.

This also points to differences between districts belonging to the same urban–rural regional type. For example, several sparsely populated rural districts in eastern Bavaria have increased their share of good employment opportunities, while equally rural districts in northeastern Germany have seen a significant decline in good employment opportunities. Among the ten districts with the highest growth in quintile five employment, only three are larger cities—Darmstadt, Wolfsburg, and Ulm—while many smaller cities and even two rural districts are also included (Table A4 in the Online Appendix). However, all these districts, with the exception of Volkswagen city Wolfsburg, are located in the south of the country. This illustrates again how growth in highly paid jobs in Germany was not restricted to the largest metropolitan areas but also took place in high-tech locations in well-connected smaller cities and on the outskirts of metropolitan areas. Out of the ten districts with the least favorable employment evolution in quintile five, eight are rural or very rural districts, along with the two cities of Gera and Erfurt, all of which are located in the former GDR (Table A5 in the Online Appendix).

However, when the proportion of employment in quintile five across districts in 2019 is examined, rather than the changes in employment, the picture is somewhat different. Figure 5b shows the share of employment in the top quintile of total employment in 2019 compared to the national average in the same year (~16%). The (dark) red districts have a lower share of quintile five employment than the national average, while the (dark) blue districts have a higher share than the national average. Similar to the above-average growth of employment, the districts with the highest shares of quintile five jobs are concentrated in the south, in the cities of Erlangen (40%) and Ingolstadt (38%), and in the suburban district outside Munich (38%). As found in the regression model, districts with a low proportion of quintile five employment are spread across eastern and western Germany, with a notable concentration in the peripheral districts of northern Germany. The three districts with the lowest proportion of quintile five employment are Nordfriesland (7%), located in the very north of Germany; Ludwigshafen/Frankenthal/Speyer (9%) in Rhineland-Palatinate; and Cloppenburg (9%) in Lower Saxony.

This analysis of individual districts also illustrates the findings of our regression analysis, which indicated that the urban–rural divide is more pronounced in terms of the share of highly paid employment. All ten districts with the highest quintile five employment share are urban districts. This includes three of Germany’s largest cities, Stuttgart, Munich and Frankfurt, as well as several of the districts in their respective metropolitan areas. (Appendix, Table A6). Conversely, most of the ten districts with the lowest proportion of employment in highly paid occupations are rural areas, with the exception of one larger city in the Ruhr area, Bottrop (Online Appendix, Table A7).

## Conclusion

This article critically examined the active debate on the urban–rural divide in Germany. Amid so far mixed empirical evidence on the actual extent of such a divide, we have focused on changes in the quality of employment at the district level and analyzed divergences between urban and more rural districts in Germany, both in terms of long-term changes in the employment structure as well as the current distribution of employment. In addition, we have analyzed whether urban–rural divides in the employment structure are consistent across two other well-established lines of geographic polarization in Germany, namely east–west and north–south divides.

Four key empirical findings emerge from our analysis. First, we find—on an aggregate level—significant differences between urban and rural districts when it comes to the quality of their employment opportunities. While employment change follows a similar upgrading trend across all types of districts, it is far stronger in the more urban districts. We also find marked differences in the current employment structure of urban and rural districts, lending support for the claim of thriving urban areas and left-behind rural areas. Second, we observe that the clear-cut differences in employment changes between urban and rural regions do not fully persist within different larger regions of Germany. While the strength of employment upgrading in the northwest and, to a lesser extent, the east aligns with the general urban–rural pattern we observed, upgrading in the southern districts has been more equally spread across urban and rural districts. Third, when comparing these different geographic divides, we find that the north–south and east–west divides are more significant in explaining changes in the number of high-wage jobs, while the urban–rural divide is more significant in explaining the current share of high-wage employment in a district. Fourth, we describe how the presence of various geographic divides leads to heterogeneity within types of urban and rural districts. While some smaller cities and urban districts, mostly in southern Germany, have seen a growth in well-paid employment possibilities that was much higher than the national average, there are urban and rural districts, mostly in eastern and northern Germany, that are left behind by national upgrading trends.

There are some limitations to our approach that should be addressed in future research. First, our analysis does not include civil servants and the self-employed, as they are not registered in the SIAB data. Although these groups account for a small share of total employment (14.8% in 2019; Federal Statistical Office of Germany 2024), changes in the number of self-employed individuals in particular could provide additional information on the quality of the local labor market structure. Second, our analyses are constrained by the aggregated occupational groups provided in the SIAB. This aggregation means we cannot account for more nuanced trends within occupational groups, such as potential variations in skill requirements for the same job across different locations. In particular, similar occupations may require higher skill levels in metropolitan areas, leading us to underestimate the extent of the skill-based divergence between urban and rural areas (Atalay et al. 2024). Third, our analysis focuses mostly on qualitative changes in the employment structure, without accounting for absolute changes in the number of jobs available in a district. However, it is possible for a district to display occupational upgrading and a decrease in overall employment simultaneously. Another limitation is that we focus solely on employment while neglecting its flip side: unemployment. Our analysis does not indicate whether the observed job losses in the lower and middle quintiles are a result of widespread occupational upgrading or if occupational upgrading only benefits certain parts of the workforce while leaving others unemployed. Lastly, our analysis is based on the assumption that jobs in occupations with higher median wages will also guarantee better life chances for people wherever they live—which neglects that the benefits of good employment might at least be partially diminished in some economically thriving districts by the higher costs of living. While in some regions, such as Munich, the disproportionally high cost of living is compensated by significantly higher salaries, this is not the case for other regions, such as Hamburg, Frankfurt, or Stuttgart (Henry et al. 2023; Wirtschaftswoche 2023). All these aspects offer ample possibilities for future investigation of regional inequalities.

Despite these limitations, our findings make several contributions to the debate on urban–rural divides and spatial inequality. First of all, our analysis extends our knowledge of urban–rural divides outside the Anglo-Saxon context by highlighting that even in a decentralized and polycentric country such as Germany, opportunity gaps between urban and rural places are a reality. This supports the numerous studies that see urban–rural divides in attitudes or voting behavior as being based on economic inequality between these places (Rodríguez-Pose 2018). Yet the findings of our analyses of individual districts also highlight that there is considerable variance within broader urban–rural categorizations. We should thus be careful in assuming a linear relationship between a place’s urbanity and its economic opportunities.

Additionally, our analyses underline that disparities between districts are shaped by geographic divides beyond the urban–rural divide. These specific lines of geographic divides vary across countries, as they depend on a country’s historical, cultural, and economic development. Consequently, the possible alignment of these geographic divides may result in a distinct structure of regional inequality in each country. This is illustrated by the case of eastern Germany, where most districts are still coping with large structural changes, such as the loss of key industries, that occurred after the collapse of the GDR. Moreover, a large proportion of Germany’s (very) rural districts are found in eastern Germany and thus face the specific challenges associated with low population density, such as brain drain and poor infrastructure. Although the results of our analyses on regional employment change cannot be easily transferred to other national contexts, we showcase that we need to think about geographic divides in a more comprehensive way in order to improve our understanding of spatial inequality.

Methodologically, we demonstrate how examining the quality of employment opportunities can offer valuable insights into the discussion and provide both long-term and spatially detailed indicators for future research, capturing a more tangible aspect of regionally diverging life chances. In addition, by focusing on long-term change rather than momentary snapshots, our research connects more directly to debates about the political and social consequences of sustained economic decline (McKay 2019). Through this approach, our study contributes to an emerging scholarship that examines regional disparities with greater geographic precision and nuance (Arzheimer and Bernemann 2023).

## Footnotes

1 Data access was provided via a scientific use file supplied by the Research Data Centre of the German Federal Employment Agency at the Institute for Employment Research.

2 The indicator refers to the year 2019, as values for earlier years are not available. Thus, the analysis refers to the evolution of districts classified as urban or rural in 2019. However, there are only a few exceptions where districts gained or lost so many inhabitants that they would have been classified in a different urban–rural category at the beginning of the observation period. Since the number of districts used by the BBSR is not the same as the aggregated districts used by the SIAB, some harmonization was necessary. Only in 12 cases was the indicator not the same for two merged districts. In this case, the aggregated districts were assigned to the category of the district with the larger population if the original indicators for both districts were next to each other. In the rare case where the aggregated districts were two categories apart, the aggregated district was placed in the category in between.

3 Daily wages were inflation adjusted and transformed to euros in 2015 (Dauth and Eppelsheimer 2020).

4 The 126th category “other occupations” was excluded from the analysis due to its imprecision.

5 Quintiles contain as close as possible to 20% of total employment in the year of the ranking. Due to the different sizes of occupation groups, the true quintile sizes can, however, deviate by 2–3% from 20%.

6 An illustration of the employment shares across job-quality quintiles at the beginning of our study period in 1993 can be found in Fig. A3 in the Online Appendix. However, due to the reranking, the change in employment shown in Fig. 2 cannot be directly deduced from the employment levels in 1993 and 2019.

## References

[CR1] Arzheimer, Kai, and Theresa Bernemann. 2023. “Place” does matter for populist radical right sentiment, but how? Evidence from Germany. *European Political Science Review* 16(2):167–186

[CR2] Atalay, Enghin, Sebastian Sotelo and Daniel Tannenbaum. 2024. The geography of job tasks. *Journal of Labor Economics* 42(4):979–1008.

[CR3] Autor, David. H. 2019. Work of the past, work of the future. *AEA Papers and Proceedings* 109:1–32.

[CR4] Autor, David. H., and D. Dorn. 2013. The growth of low-skill service jobs and the polarization of the US labor market. *American Economic Review* 103(5):1553–1597.

[CR5] Autor, David. H., Lawrence F. Katz and Melissa S. Kearney. 2008. Trends in U.S. wage inequality: revising the revisionists. *The Review of Economics and Statistics* 90(2):300–323.

[CR6] Backes, Annika, and Steffen Müller. 2024. Import shocks and voting behavior in Europe revisited. *European Journal of Political Economy* 83:102528.

[CR7] Belitz, Heike, Martin Gornig and Alexander Schiersch. 2019. Produktivität: Unterschiede zwischen Stadt und Land wichtiger als zwischen Ost und West. *DIW-Wochenbericht* 86(43):793–799.

[CR8] Bollmann, Ralph, and Inge Kloepfer. 2018. Deutschland, ein geteiltes Land. FAZ.NET. Retrieved from https://www.faz.net/aktuell/wirtschaft/deutschland-ein-geteiltes-land-15456747.html (Accessed: 10 May 2023).

[CR9] Braml, Martin, and Gabriel Felbermayr. 2018. Regionale Ungleichheit in Deutschland und der EU: Was sagen die Daten? *ifo Schnelldienst* 71(7):36–49.

[CR10] Brown, David L., and Kai A. Schafft. 2011. Rural people and communities in the 21st century: Resilience and transformation. Malden: Polity Press.

[CR66] Bundesinstitut für Bau-, Stadt- und Raumforschung (BBSR). 2019. Raumgliederungen auf Regionsbasis: Siedlungsstrukturelle Kreistypen. https://www.bbsr.bund.de/BBSR/DE/forschung/raumbeobachtung/Raumabgrenzungen/deutschland/regionen/siedlungsstrukturelle-regionstypen/siedlungsstruk-regionstypen-2019.csv?__blob=publicationFile&v=3.

[CR11] Colantone, Italo, and Piero Stanig. 2018. Global competition and Brexit. *American Political Science Review* 112(2):201–218.

[CR12] Dauth, Wolfgang. 2014. Job Polarization on local labor markets. *IAB-Discussion Paper* 18.

[CR13] Dauth, Wolfgang, and Johann Eppelsheimer. 2020. Preparing the Sample of Integrated Labour Market Biographies (SIAB) for Scientific Analysis: A Guide. *Journal for Labour Market Research* 54(1):10.

[CR65] Dauth, Wolfgang, Sebastian Findeisen and Jens Südekum. 2017. Trade and manufacturing jobs in Germany. *DICE Discussion Paper *242, Düsseldorf Institute for Competition Economics (DICE).

[CR14] Dauth, Wolfgang, Sebastian Findeisen, Enrico Moretti and Jens Südekum. 2022. Matching in cities. *Journal of the European Economic Association* 20(4):1478–1521.

[CR15] Davis, Donald R., and Jonathan I. Dingel. 2020. The comparative advantage of cities. *Journal of International Economics* 123:103291.

[CR16] Davis, James C., Anil Rupasingha, John Cromartie and Austin Sanders. 2022. Rural America at a Glance. Economic Research Service U.S. Departement of Agriculture. https://www.ers.usda.gov/webdocs/publications/105155/eib-246.pdf?v=5251.5 (Accessed: 8 Jan. 2024).

[CR63] Diamond, Rebecca. 2016. The Determinants and Welfare Implications of US Workers’ Diverging Location Choices by Skill: 1980-2000. A*merican Economic Review *106(3):479–524.

[CR17] Dienel, Christiane. 2004. Abwanderung aus Ostdeutschland – vom Wendephänomen zum langfristigen Trend? In *Problemfall Deutsche Einheit*, eds. Rainer Hufnagel and Titus Simon, 93–110. Wiesbaden: VS Verlag für Sozialwissenschaften.

[CR18] Diermeier, Matthias. 2020. Ist mehr besser? Politische Implikationen der disparaten Daseinsvorsorge in Deutschland. *Zeitschrift für Politikwissenschaft* 30(4):539–568.

[CR19] Dijkstra, Lewis, Hugo Poelman and Andrés Rodríguez-Pose. 2020. The geography of EU discontent. *Regional Studies* 54(6):737–753.

[CR20] Essletzbichler, Jürgen, Franziska Disslbacher and Mathias Moser. 2018. The victims of neoliberal globalisation and the rise of the populist vote: A comparative analysis of three recent electoral decisions. *Cambridge Journal of Regions, Economy and Society* 11(1):73–94.

[CR21] Eurofund. 2024. The changing structure of employment in the EU: Annual Review 2023. *Eurofound Research Paper*. Luxembourg: Publications Office of the European Union.

[CR22] Federal Statistical Office of Germany. 2024. Erwerbstätige: Deutschland, Jahre (bis 2019), Stellung im Beruf, Geschlecht. https://www-genesis.destatis.de/datenbank/online/statistic/12211/table/12211-9005/search/s/MTIyMTEgdW5kIGVyd2VyYnN0JUMzJUE0dGlnZQ== (Accessed: 14 Nov. 2024).

[CR23] Fink, Philipp, Martin Hennicke and Heinrich Tiemann. 2019. Ungleiches Deutschland – Sozioökonomischer Disparitätenbericht 2019. Friedrich-Ebert-Stiftung.

[CR64] Florida, Richard. 2017. *The New Urban Crisis: How Our Cities Are Increasing Inequality, Deepening Segregation, and Failing the Middle Class-and What We Can Do About It*. New York: Basic Books.

[CR24] Frieden, Immo, Andreas Peichl and Paul Schüle. 2023. Regional income inequality in Germany. *EconPol Forum* 24(2):50–55.

[CR25] Frodermann, Corinna, Andreas Ganzer, Alexandra Schmucker and Philipp vom Berge. 2021a. Factually anonymous Version of the Sample of Integrated Labour Market Biographies (SIAB-Regionalfile)—Version 7519 v1.

[CR26] Frodermann, Corinna, Andreas Ganzer, Alexandra Schmucker and Philipp vom Berge. 2021b. Sample of Integrated Labour Market Biographies Regional File (SIAB-R) 1975–2019. Data-Report.

[CR27] Fuest, Clemens, and Lea Immel. 2019. Ein zunehmend gespaltenes Land? – Regionale Einkommensunterschiede und die Entwicklung des Gefälles zwischen Stadt und Land sowie West- und Ostdeutschland. *ifo Schnelldienst* 72(16):19–28.

[CR28] Goos, Maarten, and Alan Manning. 2007. Lousy and lovely jobs: The rising polarization of work in Britain. *The Review of Economics and Statistics* 89(1):118–133.

[CR29] Gordon, Ian R. 2018. In what sense left behind by globalisation? Looking for a less reductionist geography of the populist surge in Europe. *Cambridge Journal of Regions, Economy and Society *11(1):95–113.

[CR30] Haffert, Lukas. 2022. *Stadt, Land, Frust*. München: C.H. Beck.

[CR31] Henry, Goecke, Henger Ralph, Schröder Bjarne, Schröder Christoph and Wendt Jan Marten. 2023. Regionaler Preisindex für Deutschland – ein neuer Erhebungsansatz mit Big Data. Gutachten in Zusammenarbeit mit dem Bundesinstitut für Bau-, Stadt- und Raumforschung (BBSR) im Bundesamt für Bauwesen und Raumordnung (BBR), Köln. https://www.iwkoeln.de/studien/henry-goecke-ralph-henger-bjarne-schroeder-christoph-schroeder-jan-marten-wendt-regionaler-preisindex-fuer-deutschland-ein-neuer-erhebungsansatz-mit-big-data.html (Accessed: 10 Nov. 2023).

[CR32] Immel, Lea, and Andreas Peichl. 2020. Regionale Ungleichheit in Deutschland: Wo leben die Reichen und wo die Armen? *ifo Schnelldienst* 73(5):43–47.

[CR33] Jennings, Will, and Gerry Stoker. 2016. The bifurcation of politics: Two Englands. *The Political Quarterly* 87(3):372–82.

[CR34] Johnson, Kenneth M., and Daniel T. Lichter. 2019. Rural depopulation: Growth and decline processes over the past century. *Rural Sociology *84(1):3–27.

[CR35] Kemeny, Tom, and Michael Storper. 2020. The fall and rise of interregional inequality: Explaining shifts from convergence to divergence. *Scienze Regionali* 2:175–198.

[CR36] Konietzka, Dirk, and Yevgeniy Martynovych. 2022. Die These der räumlichen Polarisierung in der neuen Klassengesellschaft. Ein empirischer Beitrag zur sozialen Spaltung von Stadt und Land. *Kölner Zeitschrift für Soziologie und Sozialpsychologie* 74:169–202.

[CR37] Konietzka, Dirk, and Yevgeniy Martynovych. 2023. The spatial dimension of social stratification in Germany—Are social class differentials in place of residence increasing? *Social Sciences* 12(6):326.

[CR38] Krugman, Paul. 1991. Increasing returns and economic geography. *Journal of Political Economy* 99(3):483–499.

[CR39] Küpper, Patrick, and Jan Cornelius Peters. 2019. *Entwicklung regionaler Disparitäten hinsichtlich Wirtschaftskraft, sozialer Lage sowie Daseinsvorsorge und Infrastruktur in Deutschland und seinen ländlichen Räumen*. Research Report 66. Thünen Report. Braunschweig: Johann Heinrich von Thünen-Institut.

[CR40] Lammers, Konrad. 2003. Süd-Nord-Gefälle in West- und Ostdeutschland? *Wirtschaftsdienst* 83(11):736–739.

[CR41] Lang, Thilo, and Lukas Vonnahme. 2020. Hidden Champions in Ländlichen Räumen. In *Land in Sicht. Ländliche Räume in Deutschland: Zwischen Prosperität und Peripherisierung*, Schriftenreihe, eds. Christian Krajewski and Claus-Christian Wiegandt, 214–227. Bonn: Bundeszentrale für politische Bildung.

[CR42] Mau, Steffen. 2024. *Ungleich vereint: Warum der Osten anders bleibt*. 1st. edition suhrkamp Sonderdruck. Berlin: Suhrkamp.

[CR43] Mau, Steffen, Thomas Lux and Julian Heide. 2024. Ost- und Westdeutsche für immer? Zu Wahrnehmungen von Unterschieden und Konflikten zwischen Ost- und Westdeutschen. *Kölner Zeitschrift für Soziologie und Sozialpsychologie *76:1–23.

[CR44] McKay, Lawrence. 2019. ‘Left behind’ people, or places? The role of local economies in perceived community representation. *Electoral Studies* 60:102046.

[CR45] Moretti, Enrico. 2013. *The new geography of jobs*. Reprint. Boston, MA: Harper Business.

[CR46] Oberst, Christian A., Hanno Kempermann and Christoph Schröder. 2019. Räumliche Entwicklung in Deutschland. In *Die Zukunft Der Regionen in Deutschland: Zwischen Vielfalt und Gleichwertigkeit*, eds. Michael Hüther, Jens Südekum and Michael Voigtländer. IW-Studien – Schriften zur Wirtschaftspolitik aus dem Institut der Deutschen Wirtschaft.

[CR47] Oesch, Daniel. 2013. *Occupational change in Europe: How technology and education transform the job structure*. Oxford: Oxford University Press.

[CR48] Oesch, Daniel, and Giorgio Piccitto. 2019. The polarization myth: Occupational upgrading in Germany, Spain, Sweden, and the UK, 1992–2015. *Work and Occupations* 46(4):441–469.

[CR49] Patana, Pauliina. 2020. Changes in local context and electoral support for the populist radical right: Evidence from Finland. *Party Politics* 26(6):718–729.

[CR50] Patana, Pauliina. 2022. Residential constraints and the political geography of the populist radical right: Evidence from France. *Perspectives on Politics* 20(3):842–859.

[CR51] Pike, Andy, Vincent Béal, Nicolas Cauchi-Duval, Rachel Franklin, Nadir Kinossian, Thilo Lang, Tim Leibert, Danny MacKinnon, Max Rousseau, Jeroen Royer, Loris Servillo, John Tomaney and Sanne Velthuis. 2023. ‘Left behind places’: A geographical etymology. *Regional Studies* 58(6):1167–1179.

[CR52] Reckwitz, Andreas. 2019. *Das Ende der Illusionen: Politik, Ökonomie und Kultur in der Spätmoderne.* Berlin: Suhrkamp.

[CR53] Ritschl, Albrecht. 1995. Aufstieg und Niedergang der Wirtschaft der DDR: Ein Zahlenbild 1945–1989. *Economic History Yearbook* 36(2):11–46.

[CR54] Rodríguez-Pose, Andrés. 2018. The revenge of the places that don’t matter (and what to do about it). *Cambridge Journal of Regions, Economy and Society* 11(1):189–209.

[CR55] Roesler, Jörg. 1994. Privatisation in Eastern Germany—Experience with the Treuhand. *Europe-Asia Studies* 46(3):505–517.

[CR56] Röhl, Klaus-Heiner. 2018. Regionale Konvergenz: Der ländliche Raum schlägt sich gut. *Wirtschaftsdienst* 98(6):433–438.

[CR57] Rosés, Joan R., and Nikolaus Wolf. 2021. Regional growth and inequality in the long-run: Europe, 1900–2015. *Oxford Review of Economic Policy* 37(1):17–48.

[CR58] Sixtus, Frederick, Manuel Slupina, Sabine Sütterlin, Julia Amberger and Reiner Klingholz. 2019. *Teilhabeatlas Deutschland: ungleichwertige Lebensverhältnisse und wie die Menschen sie wahrnehmen. *Berlin: Berlin-Institut für Bevölkerung und Entwicklung.

[CR59] Südekum, Jens, Wolfgang Dauth and Sebastian Findeisen. 2017. Verlierer(-regionen) der Globalisierung in Deutschland: Wer? Warum? Was tun? *Wirtschaftsdienst* 97(1):24–31.

[CR60] Wirtschaftswoche. 2023. Ranking 2023: In diesen Städten wohnen die Deutschen mit den höchsten Einkommen. November 7, 2023. https://www.wiwo.de/finanzen/steuern-recht/ranking-2023-in-diesen-staedten-wohnen-die-deutschen-mit-den-hoechsten-einkommen/29483042.html (Accessed: 2 Feb. 2024).

[CR61] Wolf, André. 2016. Das wirtschaftliche Süd-Nord-Gefälle in Deutschland: Aktuelle Befunde und Ursachen. *HWWI Policy Paper* 99.

[CR62] Wright, Erik. O., and Rachel E. Dwyer. 2003. The patterns of job expansions in the USA: A comparison of the 1960s and 1990s. *Socio-Economic Review* 1(3):289–325.

